# Orthogonally polarized dual-wavelength Nd:GdVO_4_/Nd:YVO_4_ laser at 1341 and 1342 nm with adjustable power ratio

**DOI:** 10.1371/journal.pone.0317875

**Published:** 2025-02-07

**Authors:** Hao Yu, Yongliang Li, Fahad Moazzam, Lin Lin, Bo Gao

**Affiliations:** School of Opto-electronics Engineering, Changchun University of Science and Technology, Changchun, China; Rutgers University Newark, UNITED STATES OF AMERICA

## Abstract

A continuous-wave (CW) orthogonally polarized dual-wavelength (OPDW) Nd:GdVO_4_/Nd:YVO_4_ laser at 1341 and 1342 nm on the ^4^F_3/2_ →^4^I_13/2_ transition was realized using in-band laser diode (LD) pumping with tunable wavelength from 909.40 to 915.02 nm for the first time. The operating temperature of the LD and the position of the pump beam waist were optimized to achieve high efficiency and balanced output powers of the OPDW laser. The OPDW laser at 1341 and 1342 nm was obtained with the highest total output power of 6.15 W and the power ratio of 1:1. The highest total slope efficiency and total optical-to-optical conversion efficiency with respect to the absorbed pump power at 913.61 nm were 34.9% and 32.0%, respectively. The OPDW laser at 1341 and 1342 nm have important application prospects in the fields of laser medicine, scientific research and terahertz radiation.

## 1. Introduction

The dual-wavelength lasers have broad application prospects in many fields such as lidar [1], medicine [2], optical holography [3, 4], precision laser spectroscopy [5], self-sensing metrology [6, 7], ultraviolet and visible laser generation by sum-frequency mixing [8, 9] and terahertz radiation generated by difference frequency technology [10–12]. For example, the dual wavelength lasers can be used in medical imaging techniques, such as optical coherence tomography, which uses the orthogonal polarization characteristics to provide high-resolution images of biological tissues, help doctors make diagnoses, and improve the sensitivity and accuracy of detection [2]. The dual wavelength can produce the frequency range of 0.1–3 THz, which falls into the very attractive spectral range with unique properties and promising potential for THz imaging, sensing, and THz spectroscopy applications [10]. In addition, the CW dual-wavelength lasers operating in the 1.3 μm region [13–17] have attracted a wide range of interest because of their applications in atom optical clock, sub-Doppler cooling of the silver atom and laser therapy [18–20]. These dual-wavelength lasers usually include the intracavity loss elements such as specially coated output couplers [21–26], etalons [27–29] or birefringent filters [30–32] to balance the gains and losses between the two laser wavelengths. However, one of the main difficulties with these dual-wavelength lasers was that the ratio of output powers between the two transition lines could be adjusted. To solve these problems, an effective solution was to replace a single gain medium with a compound gain medium. At present, there are three methods to adjust the ratio of output powers of two laser wavelengths. The first method is to change the axial position of the waist of pump beam in the composite gain medium [33]. The second method is to use a wedged-bonded gain medium and then changes the lateral position of the pump waist within the gain medium [34]. The third method is to adjust the operating temperature of the diode and then make the pump wavelength shift [35]. Using the three methods above, the dual-wavelength lasers operating at 1.06 μm on the ^4^F_3/2_ →^4^I_11/2_ transition had been reported for the compound gain medium [33–35]. In the present, we will propose a new method to control the output power ratio of the OPDW laser. In this work, a CW OPDW Nd:GdVO_4_/Nd:YVO_4_ laser at 1341 and 1342 nm on ^4^F_3/2_ →^4^I_13/2_ transition was realized by pumping with tunable wavelength from 909.40 to 915.02 nm. The ratio of output powers of the OPDW laser could be controlled by adjusting the operating temperature of the LD or the pump waist position. The highest total output power of the 1341 and 1342 nm OPDW laser reached 6.15 W with the power ratio of 1:1, and the corresponding total slope efficiency and total optical-to-optical conversion efficiency with respect to the absorbed pump power at 913.61 nm were 34.9% and 32.0%, respectively.

## 2. Experimental setup

**Fig 1:**
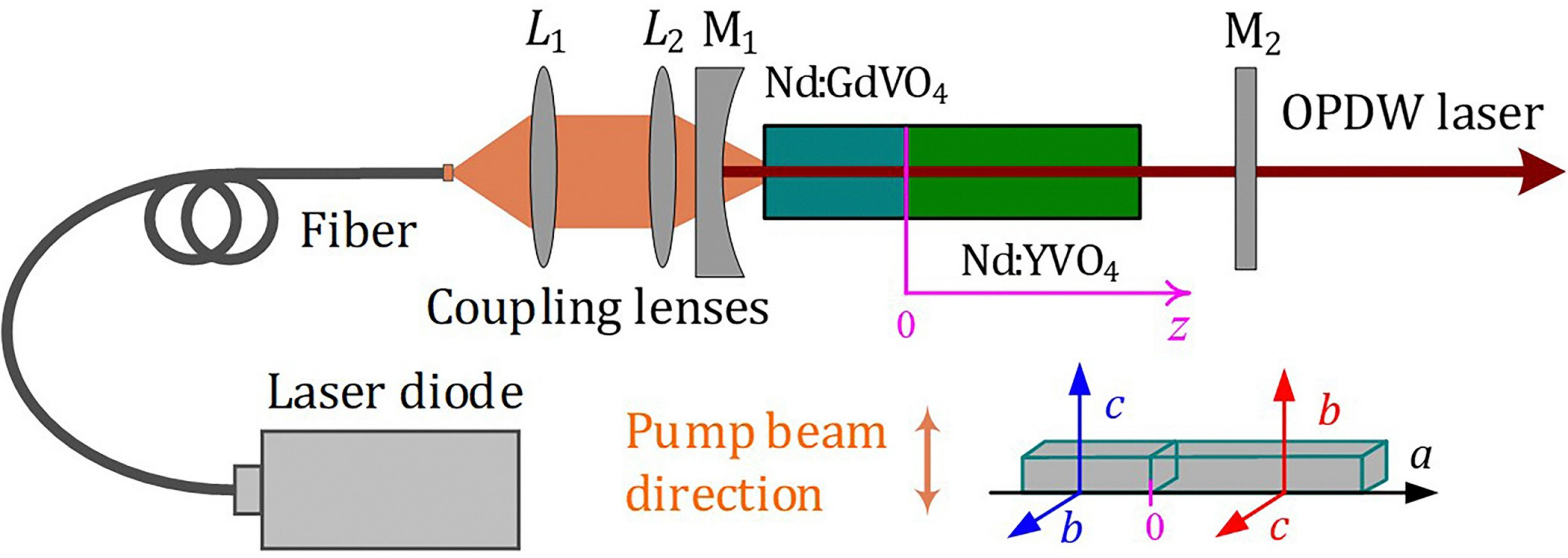
Schematic setup for the laser experiment. Inset: the arrangement of the Nd:GdVO_4_ and Nd:YVO_4_ composite crystals.

A schematic setup for the OPDW Nd:GdVO_4_/Nd:YVO_4_ laser at 1341 and 1342 nm is shown in Fig 1. A single fiber coupled laser diode (LD, BWT Ltd) with a maximum output of 30 W was used as the pump source. The core diameter of the fiber was 400 μm, the numerical aperture (NA) was 0.22 and the value of the M^2^ factor was 45. The main reason for choosing LD emitted in this band was that Nd:GdVO_4_ [36] and Nd:YVO_4_ [37] have absorption peaks at 912 and 914 nm, respectively. The temperature of the LD was cooled by the thermoelectric cooler. The LD central wavelength could be linearly tuned from 909.40 nm to 915.02 nm when its working temperature was increased from the lowest of 25°C to the highest of 38°C. The main reason that the LD output wavelength shifts with temperature is that the temperature change will cause the thermal expansion of the semiconductor material in the LD and the change of the refraction coefficient, thus affecting the LD output wavelength. The increase in temperature will reduce the band gap width of the semiconductor material, resulting in less energy released by the electronic transition. According to the inverse relationship between wavelength and energy, the LD output wavelength will be longer [38].

The pump beam was focused into the laser crystals through a 1:1 coupling lens system (*L*_1_ and *L*_2_). The two identical convex lenses *L*_1_ and *L*_2_ have a focal length of 50mm, which were antireflection (AR) coated at 910–914 nm. The flat-concave mirror (M_1_) with the radius of curvature of -300 mm (the focal length = -150 mm) was the input coupler, which was AR coated at 910–914 nm, 1060–1065 nm, and high reflectivity (HR) coated at 1340–1343 nm. The gain medium of the OPDW laser was an *a*-cut Nd:GdVO_4_ crystal with 5 mm long and 2.0 at. % doped Nd^3+^ and an *a*-cut Nd:YVO_4_ crystal with 10 mm long and 2.0 at. % doped Nd^3+^ composite crystals. The *c*-axis of the Nd:GdVO_4_ and Nd:YVO_4_ was perpendicular to each other, thus the two σ-polarizations of the 1341 nm (*S*-wave) and 1342 nm lasers (*P*-wave) generated by the Nd:GdVO_4_ and Nd:YVO_4_ crystals, respectively, were orthogonal. The arrangement of the Nd:GdVO_4_ and Nd:YVO_4_ composite crystals is shown in the inset of Fig 1. The two crystals were individually wrapped in indium foil and mounted on water-cooled copper blocks at the temperature of 16°C. The plane mirror (M_2_) was the output coupler, which was with a transmittance of 3.5% at 1340–1343 nm and AR at 1060–1065 nm. The three output couplers (2.5, 3.5 and 5%) were used and the best performance was realize using the output coupler of 3.5%.

## 3. Results and discussion

**Fig 2:**
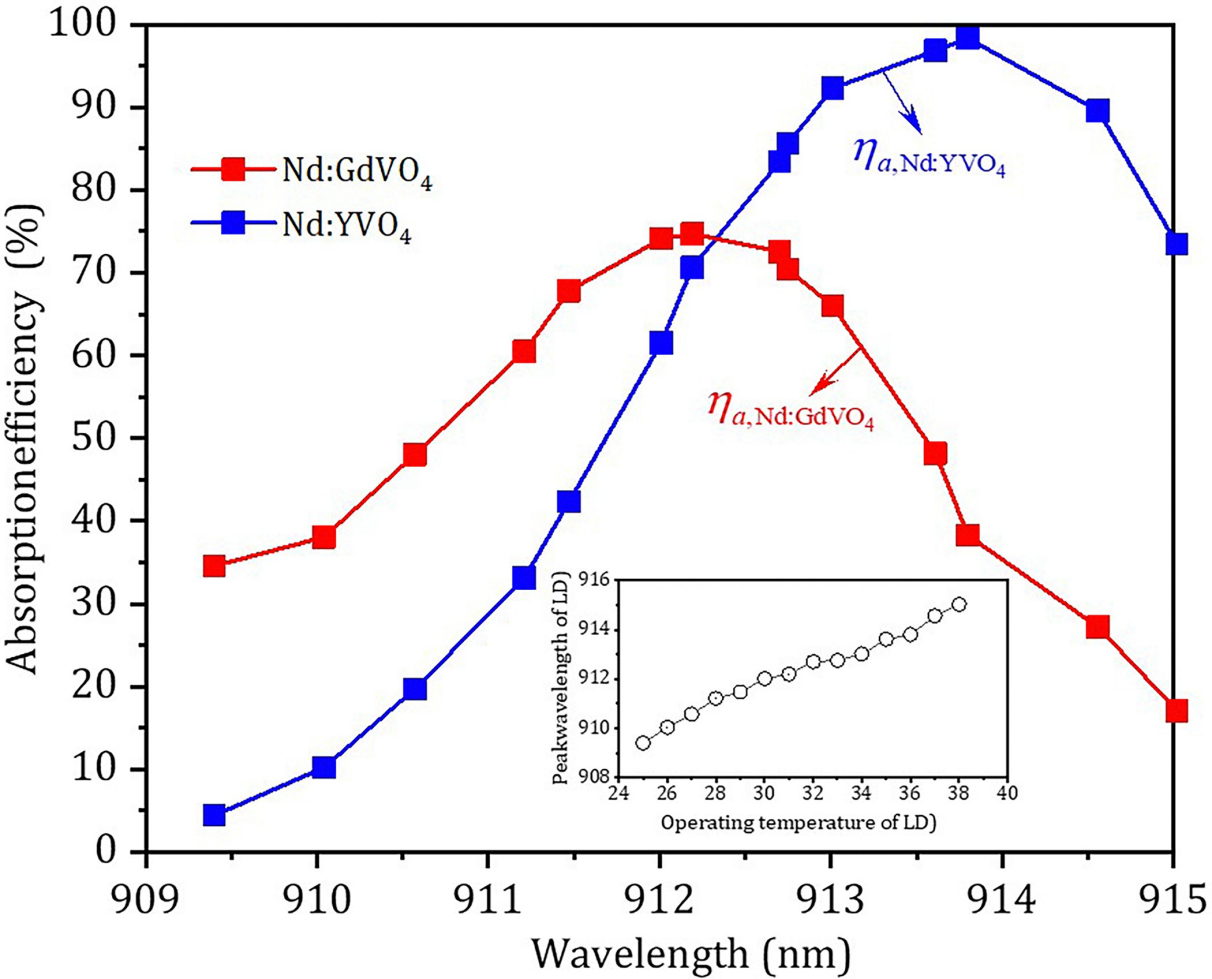
**Absorption efficiencies of the Nd:GdVO**_**4**_ **(*η***_***abs*,1341**_**) and the Nd: YV04 (*η***_***abs*,1342**_**) versus tuning wavelength of the LD, respectively.** Inset: Peak wavelength versus the operating temperature of the LD.

To balance the output power generated by Nd:GdVO_4_ and Nd:YVO_4_ crystals, the pump power was selected to be absorbed first by the Nd:GdVO_4_ crystal with the smaller absorption, and then the remaining pump power was absorbed by the Nd:YVO_4_ crystal with the larger absorption. The absorption efficiencies, *η*_*abs*,*i*_, of the Nd:GdVO_4_ and Nd:YVO_4_ crystals in region of 909.40 to 915.02 nm were measured, as shown in Fig 2, where *i* = 1341 and 1342 represents 1341 and 1342 nm wavelengths, respectively. In fact, due to the neodymium-doped vanadate absorption efficiency in the two polarization directions was different [39, 43]. In our experiment, the polarization direction of the pump beam was along the *c*-axis direction of Nd:GdVO_4_ crystal, so when the polarization direction of the pump beam was changed, the absorption ratio of the Nd:GdVO_4_ and Nd:YVO_4_ crystals was affected, resulting in the output power ratio of the OPDW laser.

To adjust the output power ratio of the OPDW laser, it can be achieved by tuning the pump wavelength or controlling the position of the pump beam waist. The measured pump peak wavelength versus the operating temperature of the LD is shown in the inset of Fig 2. It can be seen in the inset of Fig 2 that, the pump peak wavelength could be changed from 909.40 nm to 915.02 nm when the operating temperature of the LD was regulated from 25°C to 38°C. It can be seen in Fig 2 that the peak absorption efficiency of the Nd:YVO_4_ crystal was higher than that of the Nd:GdVO_4_ crystal. The Nd:GdVO_4_ crystal absorbed the pump beam first, so when the pump wavelength was adjusted to the absorption peak of the Nd:GdVO_4_ crystal, the remaining pump power (about 25%) was absorbed by the Nd:YVO_4_ crystal, resulting in a low output power generated at 1342 nm and the output powers OPDW laser could not be balanced. Similarly, when the pump wavelength was adjusted to the absorption peak of the Nd:YVO_4_ crystal, the corresponding absorption efficiency of the Nd:GdVO_4_ crystal was only about 30%, and the balanced output powers of the OPDW laser could not be achieved either. Therefore, it was necessary to properly adjust the operating temperature of the LD, not only to make full use of the pump power, but also to adjust the output power ratio generated by the two crystals in a large range. Since the emission cross-section of the Nd:GdVO_4_ crystal was slightly larger than that of the Nd:YVO_4_ crystal, the absorption efficiency of the Nd:GdVO_4_ should be less than 50%, and then the remaining pump power greater than 50% was absorbed by the Nd:YVO_4_ crystal. At this point, the optimal pumping wavelength should be close to the peak absorption efficiency of the Nd:YVO_4_ crystal.

**Fig 3:**
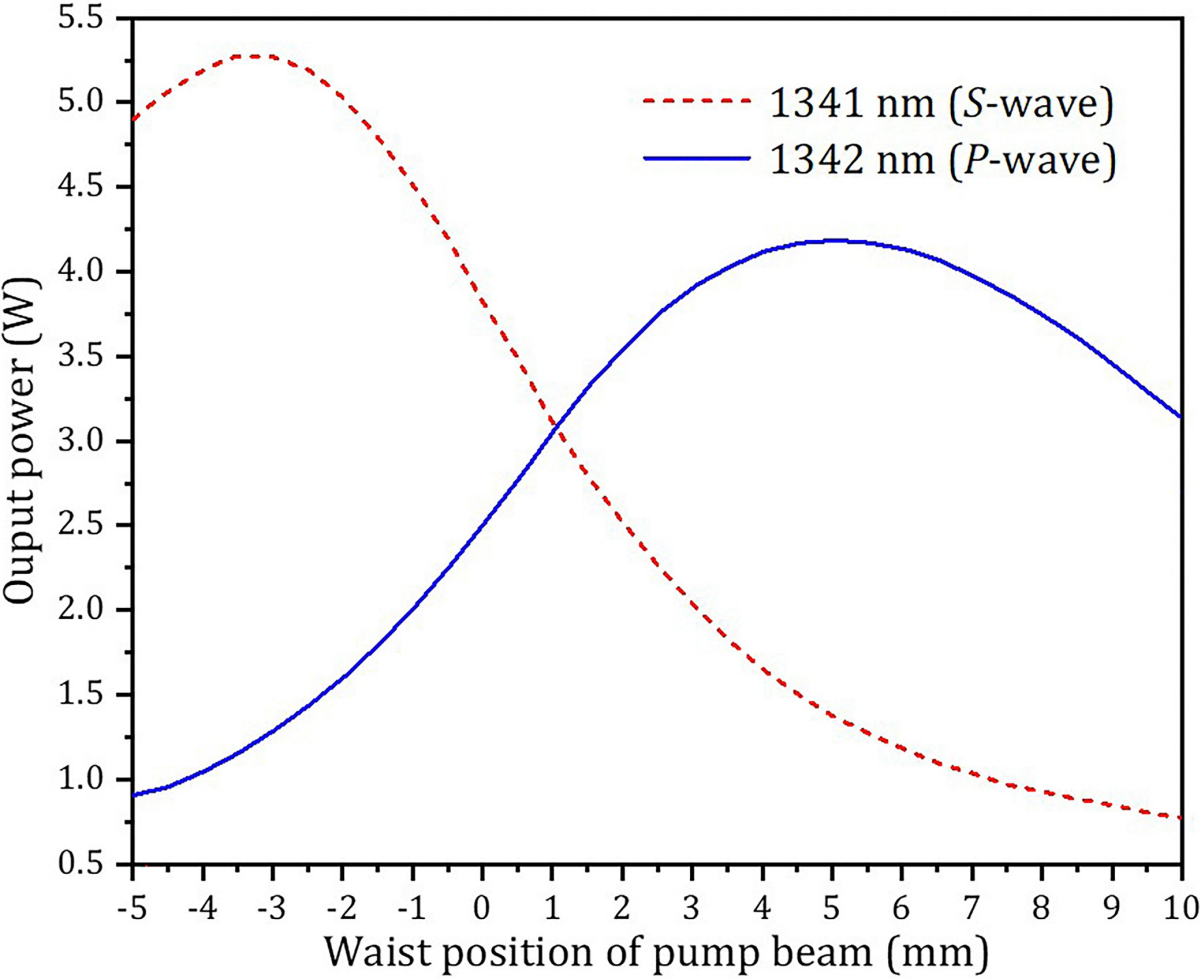
Calculated output power of *S*- and *P*-waves of the OPDW laser versus the waist position of pump beam at the LD operating temperature of 35°C.

The CW output power, *P*_*out*,*i*_, for an end-pumped four-level solid-state laser system can be expressed as [40]

$$P_{out,i}=\frac{1}{2}A_{i}T_{i}\frac{{hv}_{i}}{\sigma_{i}}\left(\frac{{2\sigma_{i}N_{i}W_{i}l}_{i}}{L_{i}}-W_{i}-\frac{1}{\tau_{i}}\right),$$ (1)

where *A* is the beam cross-section of the emission-wavelength, which was affected by the thermal lens effect of the Nd:GdVO_4_ and Nd:YVO_4_ crystals. The focal lengths of the thermal lens for the Nd:GdVO_4_ and Nd:YVO_4_ crystals were calculated by Ref. [41]. *T* is the transmittance of the output coupler, *hv* is the photon energy of the emission-wavelength, σ is the cross section of the emission-wavelength, *τ* is the fluorescence lifetime, *L* is the cavity round-trip loss, the parameter of pump beam, W, can be written as [40]

$$W_{i}=\frac{\eta_{i}p_{i}\alpha_{i}}{N_{i}{hv}_{p}l_{i}}\int \frac{e^{-\alpha_{i}z}}{n_{i}\pi \omega_{p}^{2}(z)}dz,$$ (2)

where *η* is the quantum efficiency, *p* is the incident pump power at the left end face of the Nd:GdVO_4_ crystal, *p*_1341_ = *P* and *p*_1342_ = *P* exp(–*α*_1341_ *l*_1341_) when the pump power is *P*. *α* = *σ*_*abs*_ *N*, *N* is Nd^3+^ concentration in units of ion/cm^3^ (*N*_1341_ = 2.41 ×10^20^ cm^-3^ for 2.0% doped (*N*_*ion*_) Nd:GdVO_4_ crystal and *N*_1342_ = 2.50×10^20^ cm^-3^ for 2.0% doped Nd:YVO_4_ crystal), *σ*_*abs*_ is the absorption cross section of the gain medium, *l* is the length of the gain medium, *hv*_*p*_ is the pump photon energy, M^2^ is the quality factor of the pump beam, *λ*_*p*_ is pump wavelength, *n* is the refraction index of the laser crystal. *ω*_*p*_(*z*) is the beam radius of pump wavelength, which can be given by [42]

$$\omega_{p}^{2}(z)=\omega_{p0}^{2}\left\{1+{\left[\frac{M^{2}\lambda_{p}}{n_{i}\pi \omega_{p0}^{2}}(z-z_{0})\right]}^{2}\right\},$$ (3)

where M^2^ is the quality factor of the pump beam, *λ*_*p*_ is pump wavelength, *n* is the refraction index of the gain medium, *ω*_*p*0_ is the waist radius of the pump beam, *z*_0_ is the pump waist position, and *z*_0_ = 0 was set at the interface between the Nd:GdVO_4_ and Nd:YVO_4_ crystals, *ω*(*z*) is the beam radius of the emission wavelength, which was affected by the thermal lens effect of the Nd:GdVO_4_ and Nd:YVO_4_ crystals and can be calculated by the ABCD matrix. With Eqs (1)–(3) and the parameters in the experiment: *T* = 0.035, *ν*_1341_ ≈ *ν*_1342_ = 2.24 × 10^14^ Hz, *σ*_1341_ = 3.2 × 10^−20^ cm^2^ [43], *σ*_1342_ = 6.0 × 10^−19^ cm^2^ [44], *L* = 0.05, *τ*_1341_ = 95 μs, *τ*_1342_ = 100 μs, *P* = 19.2 W, *ω*_p0_ = 190 μm, M^2^ = 45, *λ*_*p*_ = 913.61 nm, *n*_1341_ = 2.29, *n*_1342_ = 2.16, *η*_1341_ ≈ *η*_1342_ = 0.68. When the LD temperature was 35°C, ν_p_ = 3.77 × 10^14^ Hz, the laser output powers of 1341 nm (*S* wave) and 1342 nm (*P* wave) were calculated as a function of waist position of pump beam, as shown in Fig 3. It can be seen in Fig 3 that the maximum output power of 1341 nm was at *z* = -2.5, which was because the pump beam waist was exactly in the middle of the Nd:GdVO_4_ crystal, and this symmetric position was optimal for both mode matching and pump beam distribution. Similarly, the maximum output power of 1342 nm was at *z* = 5, and the corresponding pump beam waist was also in the middle of the Nd:YVO_4_ crystal.

The balanced output power was generated at *z* = 1.0 mm, when the operating temperature of the LD was 35°C. Moving the pump waist position from this point (*z* = 1.0 mm) to both sides can adjust the output power ratio of the OPDW laser.

**Fig 4:**
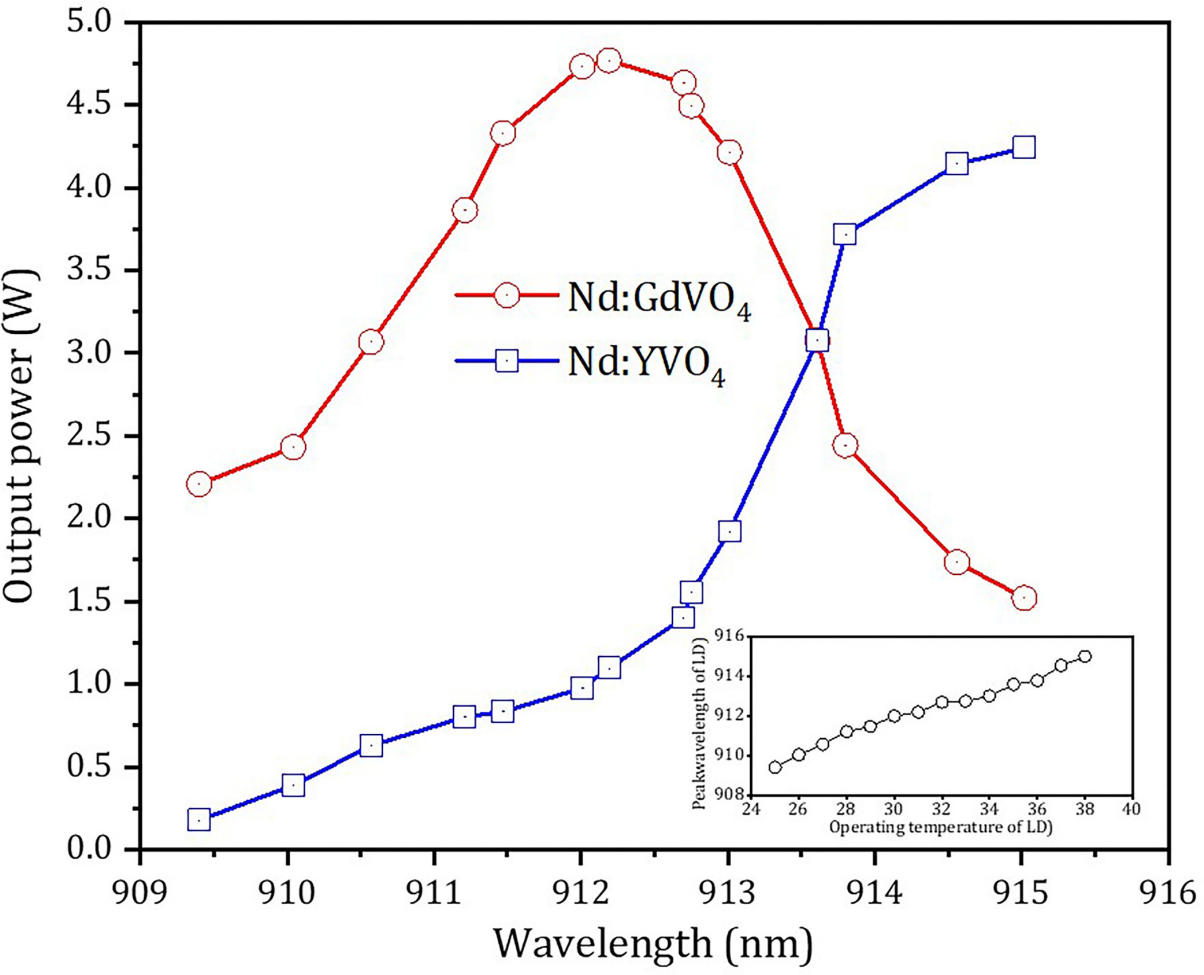
Output power of the *S*- and *P*-waves versus the output wavelength of LD when the waist position of pump beam was at *z* = 1.0 mm and the pump power was 19.2 W.

The pump waist position was retained at *z* = 1.0 mm, and the output powers of 1341 nm and 1342 nm were measured with the change of operating temperature of the LD, as shown in Fig 4. As can be seen in Fig 4, the output power of 1341 nm was greater than that of 1342 nm when the operating temperature was below 35°C. This was mainly because the Nd:GdVO_4_ crystal was excited more efficiently and involved in the main lasing process than the Nd:YVO_4_ crystal within this operating temperature range. Conversely, when the operating temperature was above 35°C, the output power characteristics of 1341 and 1342 nm were opposite.

Compared with previous dual-wavelength lasers based on physically combined or diffusion-bonded composite crystals [33, 34, 45–47], the total highest output power in this work was generated at the balanced output power, which was more conducive to OPDW laser applications, such as using difference frequency technology to achieve the most efficient terahertz radiation, and using sum-frequency technology to obtain the most efficient visible laser output.

**Fig 5:**
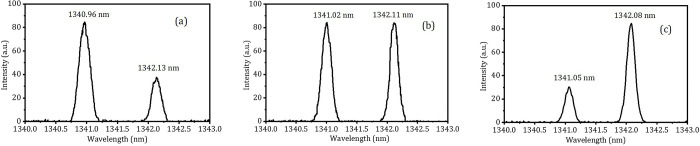
Spectra of the OPDW at 1341 and 1342 nm. (a)–(c) show the change of spectral intensity ratio between the two laser wavelengths by adjusting the operating temperature of LD. (a) *λ*_*p*_ = 912.19 nm at 31°C, (b) *λ*_*p*_ = 913.61 nm at 35°C, (c) *λ*_*p*_ = 915.02 nm at 38°C.

**Fig 6:**
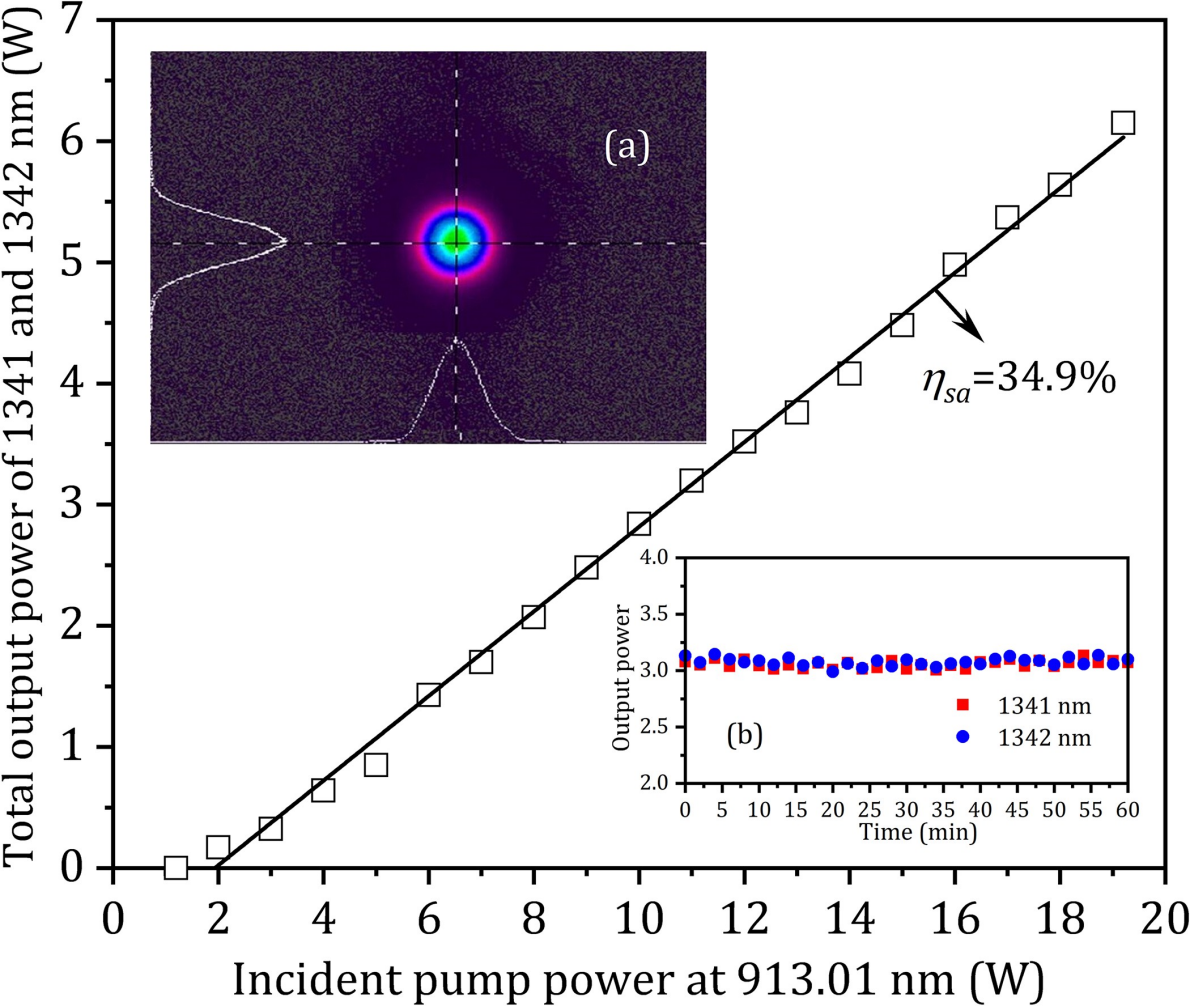
Total output power of the OPDW laser with the output power ratio of 1:1 at 1341 and 1342 nm versus the incident pump power at 913.61 nm. Inset (a): the shape of the OPDW output beam at maximum output power. Inset (b): the stability of the OPDW output powers.

The output spectra of the OPDW laser at different operating temperature of LD of 31°C, 35°C and 38°C were shown in Fig 5. The total output power of the OPDW Nd:GdVO_4_/Nd:YVO_4_ laser at 1341 and 1342 nm versus the absorbed pump power at 913.61 nm is shown in Fig 6. The maximum total output power was 6.15 W with the power ratio of 1:1 at 19.0 W (= 48.2%×19.2 W+ 51.8%×19.2 W ×98.3%) of the absorbed pump power. The corresponding slope efficiency and optical-to-optical conversion efficiency were 34.9% and 32.0%, respectively. The M^2^ factors were less than 1.12 in both directions at the maximum total output power. The inset (a) of Fig 6 shows the shape of the output beam. The M^2^ factors of the output beam were 1.07 and 1.12 in X and Y directions, respectively. The stability testing was carried out by monitoring the output powers of each wavelength with a Field-Master-GS powermeter at 10 Hz. The fluctuations for 1341 nm and 1342 nm lasers at the maximum total output power were about 2.57% and 2.62% (RMS) in 1 hour, respectively, as shown in the inset (b) of Fig 6.

## 4. Conclusion

A CW OPDW Nd:GdVO_4_/Nd:YVO_4_ laser at 1341 and 1342 nm on the ^4^F_3/2_ →^4^I_13/2_ transition was realized using in-band LD pumping with tunable peak wavelength from 909.40 nm to 915.02 nm for the first time. The operating temperature of the LD and the pump waist position were optimized to achieve high efficiency and balanced output power of the OPDW laser. The CW OPDW laser at 1341 and 1342 nm was obtained with the highest total output power of 6.15 W and the power ratio of 1:1. The highest total slope efficiency and total optical-to-optical conversion efficiency with respect to the incident pump power at 913.61 nm were 34.9% and 32.0%, respectively. We believe that the same technique presented in this paper can be applied to other composite crystals to achieve the OPDW lasers with adjustable ratio of output powers.
